# Identification of SWI2/SNF2-Related 1 Chromatin Remodeling Complex (SWR1-C) Subunits in Pineapple and the Role of Pineapple SWR1 COMPLEX 6 (*Ac*SWC6) in Biotic and Abiotic Stress Response

**DOI:** 10.3390/biom9080364

**Published:** 2019-08-13

**Authors:** Bello Hassan Jakada, Mohammad Aslam, Beenish Fakher, Joseph G. Greaves, Zeyun Li, Weimin Li, Linyi Lai, Oyekunle Adenike Ayoade, Yan Cheng, Shijiang Cao, Gang Li, Jer-Ming Hu, Yuan Qin

**Affiliations:** 1 Key Laboratory of Genetics, Breeding and Multiple Utilization of Crops, Ministry of Education, Fujian Provincial Key Laboratory of Haixia Applied Plant Systems Biology, College of Crop Science, Fujian Agriculture and Forestry University, Fuzhou 350002, China; 2 Life Science College, Fujian Agriculture and Forestry University, Fuzhou 350002, China; 3 State Key Laboratory of Ecological Pest Control for Fujian and Taiwan Crops, College of Plant Protection, Fujian Agriculture and Forestry University, Fuzhou 350002, China; 4 College of Forestry, Fujian Agriculture and Forestry University, Fuzhou 350002, China; 5 Institute of Ecology and Evolutionary Biology, National Taiwan University, Taipei 106, Taiwan; 6 State Key Laboratory for Conservation and Utilization of Subtropical Agro-Bioresources, Guangxi Key Lab of Sugarcane Biology, College of Agriculture, Guangxi University, Nanning 530004, China

**Keywords:** SWR1-C, chromatin remodeling complex, SWC6/SEF, biotic stress, pineapple

## Abstract

Chromatin remodeling complex orchestrates numerous aspects of growth and development in eukaryotes. SWI2/SNF2-Related 1 chromatin remodeling complex (SWR1-C) is a member of the SWI/SNF ATPase-containing chromatin remodeling complex responsible for the exchange of H2A for H2A.Z. In plants, SWR1-C plays a crucial role by transcriptionally regulating numerous biological and developmental processes. However, SWR1-C activity remains obscure in pineapple. Here, we aim to identify the SWR1-C subunits in pineapple. By genome-wide identification, we found a total of 11 SWR1-C subunits in the pineapple. The identified SWR1-C subunits were named and classified based on the sequence similarity and phylogenetic analysis. RNA-Seq analysis showed that pineapple SWR1-C subunits are expressed differentially in different organs and at different stages. Additionally, the qRT-PCR of pineapple SWR1-C subunits during abiotic stress exposure showed significant changes in their expression. We further investigated the functions of pineapple SWR1 COMPLEX 6 (*Ac*SWC6) by ectopically expressing it in *Arabidopsis*. Interestingly, transgenic plants ectopically expressing *AcSWC6* showed susceptibility to fungal infection and enhanced resistance to salt and osmotic stress, revealing its involvement in biotic and abiotic stress. Moreover, the complementation of mutant *Arabidopsis* *swc6* by pineapple SWC6 suggested the conserved function of SWC6 in plants.

## 1. Introduction

Epigenetics is one of the major processes that orchestrate gene regulation in plants, animals, and eukaryotes in general. It involves DNA methylation, covalent modification of DNA, and histone modifications [1]. It is also linked with DNA methylation resulting in transcriptional silencing [2,3,4]. In plants, epigenetics is essential for reproduction, the viability of seeds, development of the pollen grain, and it ensures proper regulation of genes [2]. Development of eukaryotes from embryo to mature organism is regulated by sophisticated biological processes, including chromatin remodeling [5]. Epigenetic regulation at nucleosomes is largely dependent on multiprotein covalent alteration of histones or ATP-dependent chromatin remodeling proteins. Changes in gene expressions are governed by chromatin remodeling that ensures successful developmental and physiological responses [6]. This phenomenon is regulated by ATP-dependent chromatin remodelers such as INO80, CHD, and SWI/SNF [7].

SWI2/SNF2-RELATED 1 is largely known as SWR1 chromatin remodeling complex (SWR1-C) and it is conserved throughout the eukaryotes [6,8]. SWR1-C is reported to facilitate the exchange of histone H2A with variant histone H2A.Z [9]. Exchange of H2A.Z has a great influence on gene expression due to its peculiar physicochemical characteristics [10]. H2A.Z affects nucleosome stability and changes chromatin architecture to fine tune gene expression positively or negatively [9]. H2A.Z governs transcription through modification of chromatin accessibility in the eukaryotes [11,12,13]. In yeast, fourteen subunits of the SWR1-C subunits have been identified [10]. These are SWR1 a homologue of PHOTOPERIOD INDEPENDENT AND EARLY FLOWERING (PIE1) in *Arabidopsis*, SWR1 COMPLEX2 (SWC2), SWR1 COMPLEX 3 (SWC3), SWR1 COMPLEX 5 (SWC5), SWR1 COMPLEX 6 (SWC6) which is a homologue of SERRATED LEAF AND EARLY FLOWERING (SEF) in *Arabidopsis*, SWR1 COMPLEX 7 (SWC7), ACTIN-RELATED PROTEIN 6 (ARP6), YEAST ALL1 FUSED GENE FROM CHROMOSOME 9 (YAF9), BROMODOMAIN-CONTAINING FACTOR 1 (BDF 1) which is also known as GTE8 in *Arabidopsis*, ACTIN 1 (ACT1), ACTIN-RELATED PROTEIN 4 (ARP4), SWR1 COMPLEX 4 (SWC4), RUVB LIKE PROTEIN 1 (RVB1), and RUVB LIKE PROTEIN 2 (RVB2) [4,14]. Some of the SWR1-C subunits are shared members between SWR1-C and other chromatin remodeling complexes. ACT1, ARP4, RVB1, and, RVB2, are also subunits of the INO80 chromatin-remodeling complex. Similarly, SWC4, YAF9, ACT1, and ARP4 are also subunits shared with NuA4 acetyl transferase complex [4,10].

SWR1-C subunits, such as ARP6 and SWC6, facilitate binding and histone replacement while SWC2, together with ARP6, SWC6, and SWR1, acts as a subcomplex. Similarly, they associate with each other and are required for the association of other SWR1-C subunits [10,15]. SWC2 is important for H2A.Z transfer, association, and nucleosome binding [15]. SWC6 and ARP6 are required for H2A.Z deposition, while other components such as SWC5 and YAF9 function in H2A.Z transfer but not nucleosome binding. Association of several subunits, such as BDF1, YAF9, and SWC4, requires ARP4 for their activity. Null mutation of a number of nonessential SWR1-C subunits has clearly demonstrated that H2A.Z deposition in vivo is linked to YAF9, ARP6, SWC2, and SWC6, as well as PIE1 [4]. In plants, developmental processes such as flower development, stress tolerance, immunity, DNA damage repair, and transition from the vegetative phase to reproductive phase require the action of SWR1-C [10,16,17]. SWR1 also represses anthocyanin accumulation by depositing H2A.Z at anthocyanin biosynthetic genes which negatively regulates the expression of these genes in *Arabidopsis* [18].

Loss of function of key SWR1-C subunits such as ARP6, SWC6 (SEF), or PIE1 in *Arabidopsis* is characterized by a pleiotropic phenotype affecting vegetative and reproductive phases of plant developmental processes [6]. SWC6 mutation in *Arabidopsis* triggers developmental irregularities by reducing transcript levels of MADS AFFECTING FLOWERING 4 (MAF4) and FLOWERING LOCUS C (FLC) and accelerates the expression of SUPPRESSOR OF OVEREXPRESSION OF CO1 (SOC1) which are important genes in the flowering pathway [12]. SWC6 mutants exhibit leaf serration, absence of internodes in the floral organs, bushy phenotype, and inflorescence with the irregular number of floral parts [19]. In *Arabidopsis*, *swc6* phenotype is similar to that of the *pie1* and the *arp6* mutants but *pie1* mutants show a severe phenotype compared to *arp6* and *swc6* [17,19,20,21]. Functional studies suggest the genetic association and interaction between ARP6, SWC6, and PIE1 [5,21]. Moreover, ARP6 mutation directly affects meiosis during female gametogenesis by controlling meiotic genes [22]. SWR1-C is also involved in immune response. Mutation in SEF and PIE1, core SWR1-C subunits, triggers immune responsive genes, revealing the importance of the SWR1-C complex in immunity and biotic interaction [10]. SWC6 is a conserved member of plant SWR1 complex and a homolog of SEF in *Arabidopsis* [10,19]. It is implicated in the substitution of H2A with H2A.Z variant, thus affecting chromatin structure which in turn facilitates gene expression through turning on and turning off of a number of genes [5,19]. Mutation in SWC6 in *Arabidopsis* is characterized by early flowering, leaf serration, irregular numbers of petals, and reduced apical dominance as a result of reduced expression of FLC, MAF4, and MAF5 [5,23]. SWR1-C has been well characterized in yeast, *Arabidopsis*, and humans [5].

Pineapple (*Ananas comosus* L. Merr) is among the popular exotic fruits worldwide and is a member of the *Bromeliaceae* family domesticated in northern and southern America about 6000 years ago [24,25,26,27]. It is one of the most significant tropical fruits after banana and citrus [28,29]. Pineapple fruit is well known for its great flavor, taste, and fragrance and is cultivated on a continental scale with medicinal and nutritional value [24]. It is rich in vitamins such as vitamin A, B, and C [29]. Among the *Bromeliaceae* family, only pineapple has active global trade due to its flavor. Thirty million tonnes of pineapple are annually produced in the world [29].

In this study, we have characterized the SWR1-C chromatin remodeling complex in pineapple. Although SWR1-C has been well studied in *Arabidopsis*, its investigation in pineapple has not been reported. Here, we have identified 11 SWR1-C subunits in pineapple and characterized their features using different bioinformatic methods. In addition, we investigated the expression profile of identified SWR1-C subunits in different organs and developmental stages. We further validated the function of *Ac*SWC6 during biotic and abiotic stress by ectopically expressing it in *Arabidopsis*. Finally, we complemented the *Arabidopsis* (dicot) *swc6* mutant using pineapple (monocot) *Ac*SWC6. Collectively, our results suggest that the SWR1-C subunit SWC6 has a conserved function in plants.

## 2. Materials and Methods

### 2.1. Plant Material, Growth Conditions, and Treatments

Pineapple plants were MD2 propagated by tissue culture [30] and were supplied by Haixia institute of science and technology Centre for Genomics and Biotechnology, Fujian Agriculture and Forestry University, Fuzhou, China. Plastic pots containing soil mixture (peat moss: perlite = 2:1 (*v*/*v*)) were used for pineapple plants and grown in a growth house at 30 °C temperature and 60–70 mMolL^−1^ photons m^–2^ s^−1^ with 70% humidity and 16/8 h light cycle [9,31,32]. All the *Arabidopsis* plants used in this research were in background Colombia. *Arabidopsis thaliana* (L.) Col-0; (CS60000) was referred to as wild type (WT) and the T-DNA insertional mutants *Atswc6* (SAIL_536_A05) were obtained from the *Arabidopsis* Biological Resource Center (ABRC) and confirmed by genotyping. To grow plants, *Arabidopsis* seeds were planted on a modified Hoagland’s medium in round 9 cm petri plates with 1% (*w*/*v*) sucrose and 1% bacto agar (*w*/*v*) [33]. They were then kept at 4 °C in the dark for 48 h to stratify the seeds [34] before they were transferred to growth room with 22 °C, 16 h illumination of light and 8 h of darkness under the light strength of 100 μmol m^−2^ s^−1^.

For the abiotic stress treatments, 30-day old pineapple plants were subjected to different abiotic stress of NaCl 150 mM, mannitol 350 mM, heat at 45 °C and cold at 4 °C for 24, 48, and 72 h, respectively. For *Arabidopsis* abiotic treatment, wild type, *Atswc6* mutant, *Atswc6* complemented line, and the *Ac*SWC6 overexpression line were assayed for growth on Hoagland’s medium supplemented with or without NaCl 150 mM, mannitol 300 mM, and kept in a growth room. To observe the growth phenotype photographs were taken after 4 d and 6 d and analyzed.

The biotic stress for pineapple and *Arabidopsis* was carried out as described by Seifi et al., [35] with some modifications. The necrotrophic pathogen *Sclerotinia sclerotiorum* was provided by the Haixia institute of science and Technology, Fujian Agriculture and Forestry University, Fujian China, and was cultured on 2% potato dextrose agar at a temperature of 25 to 28 °C for three days. The plants were then treated with fungus for 24 h by placing it on the adaxial surface of the leaves of the plants using the agar plug method [35,36] followed by 3,3′-diaminobenzamide (DAB) staining to detect the H_2_O_2_ activity [37].

### 2.2. Quantitative Real-Time Polymerase Chain Reaction (qRT-PCR)

Pineapple plants exposed to biotic and abiotic stresses as stated above were used for RNA isolation, followed by cDNA preparation using a standard protocol. The qPCR was done using TranStart Top Green qPCR superMix in a Bio-Rad qPCR set CFX96 Touch™ real time machine (Bio-Rad, Singapore). Real-time PCR in 20 μL volume was carried out with the following steps: for 2 min at 95 °C, then 95 °C for 10 s for 40 cycles, then 15 s at 60 °C, followed by 15 s at 72 °C. The pineapple EFIα gene was used as the internal control [38,39]. The Livak method (^−ΔΔ^CT) was used for the calculation of fold change [38,39]. The experiments were carried out in three biological and three technical replicates. Primers used in real time are listed in Supplementary Table S2.

### 2.3. RNA Seq of Pineapple

RNA from gynoecium, ovule, stamen, petal, and sepal of pineapple (MD2 variety) at various stages of development were isolated for library preparation and Illumina sequencing as previously described by Chen et al., [32]. Total RNA was isolated by Plant RNeasy Mini (Qiagen, Strasse 1, Hilden, Germany), followed by cDNA library preparation using NEBNext Ultra^TM^ RNA library prep (NEB, Ipswich, MA, USA) following standard protocols. After quality checking and sequencing, the reads were aligned to the genome of pineapple using TopHat v2.1.1 [40] with default parameters. The transcript abundance of pineapple SWR1-C subunits were calculated as fragments per kilobase of exon model per million mapped reads (FPKM) and a heatmap was generated using pheatmap, a package of R software based on the log2 (FPKM+1).

### 2.4. Phylogenetic Analysis and Identification of Homologue Sequences in Pineapple

SWR1-C subunit protein sequences of *Arabidopsis* were downloaded from TAIR (http://www.arabidopsis.org) and of *Oryza sativa* from the rice data center of China (http://www.ricedata.cn/gene/index.htm). For the identification of SWR1-C subunits in pineapple, SWR1-C keywords were used to blast against the genome of pineapple in the phytozome database (https://phytozome.jgi.doe.gov); then, we performed a search in the pineapple genome database by using the HMM profiles to carryout BLAST-P with 0.01 set as the e-value. Parameters of MUSCLE 3.7 were set at default to generate the multiple sequence alignment of SWR1-C subunits in pineapple, *Arabidopsis*, and rice. The phylogenetic trees were generated using MEGA 7 by the maximum likelihood (ML) method with default parameters and settings with exception of the bootstrap option n = 1000 plus pairwise gaps removal. The neighbor-joining (NJ) method was used to generate phylogenetic trees. ExPASy (http://web.expasy.org) was used to determine the isoelectric point (pI) and molecular weight (MW) of all the SWR1-C subunits in pineapple [41,42,43]. The details of the locus of *Arabidopsis* and rice SWR1-C subunits are provided in Supplementary Table S1.

### 2.5. Gene Structure Analysis and Chromosomal Location

For the mapping of SWR1-C subunits on the various linkage groups in pineapple, MapChart [44] was used based on their start and stop identification numbers identified from the phytozome (https://phytozome.jgi.doe.gov) database using the pineapple genome. GSDS (gene structure and display server) was used to make the exon–intron structural organization in pineapple SWR1-C [45].

### 2.6. Vector Construction

Pineapple genomic DNA was used to amplify 5058 bp of complete *Ac*SWC6 gene from start codon and without stop codon using the primers listed in Supplementary Table S2. The amplified *Ac*SWC6 was then moved to the vector pENTR/D-TOPO (Invitrogen). The positive clones were then recombined with the destination vector pGWB505 using LR recombination clonase (Invitrogen, Carlsbad, CA, USA) [9]. The final cassette was transformed into GV3101 strain of *Agrobacterium tumefaciens* and used for transformation of *Atswc6* and Col-0 using the floral dip method [46].

### 2.7. Microscopy

For live microscopy, 5 d old seedlings were mounted in the on a cover glass slide for observation using a Leica TCS SP8 microscope (Leica, Wetzlar, Germany) and imaged.

### 2.8. Statistical Analysis

Results are expressed as the means ± standard error (SE) from at least three experiments. The statistical significance was analyzed by two-tailed Student’s *t*-test.

## 3. Results

### 3.1. Identification, Phylogenetic Analysis, and Gene Structure of SWR1-C Subunits

**Figure 1:**
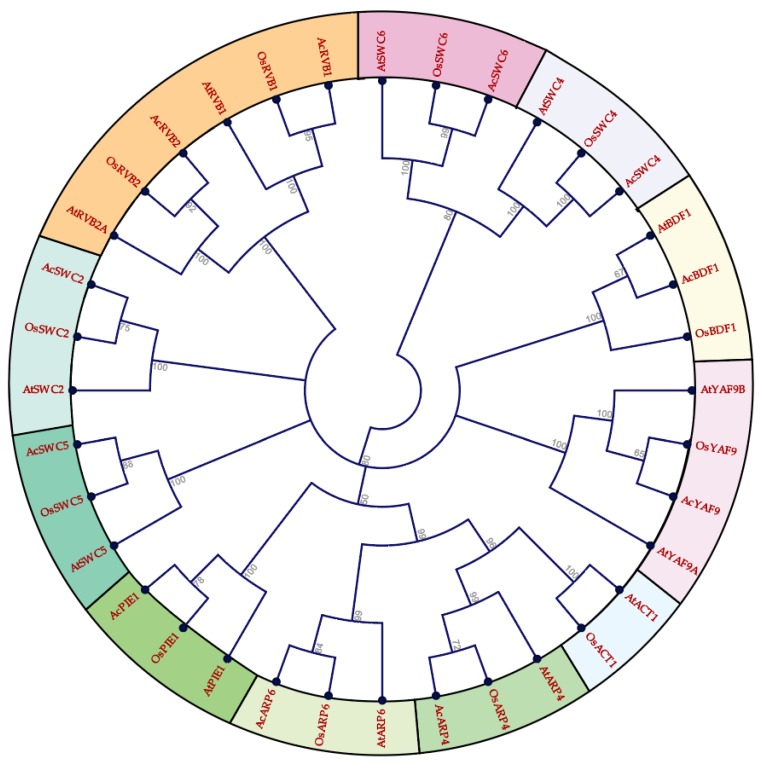
Phylogenetic tree indicating the relationships among SWI2/SNF2-Related 1 chromatin remodeling complex (SWR1-C) subunits from pineapple, rice, and *Arabidopsis*. Prefixes ‘At’, Os, and ‘Ac’ indicate SWR1-C proteins from *Arabidopsis*, *Oryza sativa*, and *Ananas comosus* respectively.

**Figure 2:**
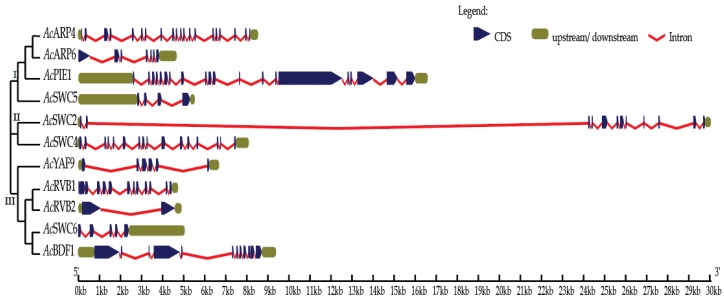
Exon–intron structure of pineapple SWR1-C subunits.

We identified 11 SWR1-C subunits in pineapple using the genome of pineapple, BLAST, HMM, and available pineapple annotation based on the SWR1-C in yeast and *Arabidopsis* [10,14,19]. To study the evolutionary and functional relationship of identified SWR1-C subunits, a phylogenetic tree of multiple species was built using the full-length amino acid sequences of pineapple, *Arabidopsis*, and rice using the maximum likelihood parameter. The pineapple SWR1-C components were then annotated based on their sequence similarity and phylogeny with other species. However, most of the individual subunits formed an independent group, with their corresponding homolog in rice and *Arabidopsis* revealing their conserved nature between monocots and dicots (Figure 1). Additionally, another phylogenetic tree was generated based on the gene structure between the individual subunits in pineapple which divided the complex into three groups: Group I, Group II, and Group III, respectively (Figure 2). Group I has four members, whereas Group III has five members. The smallest group is Group II with only two members. This suggests that structural diversity is a common phenomenon among the pineapple SWR1-C subunits which could be the reason they have nonredundant function in some biological processes.

For structural divergence of the SWR1-C subunits in pineapple, the exon–intron structures were further investigated. An exon–intron structure was constructed using the Gene Structure Display Server 2.0 (GSDS) (Figure 2). The result revealed that among the SWR1 complex subunits, only *Ac*SWC2 and *Ac*BDF1 have the same numbers of exons and introns, while other subunits of the complex exhibit variation of exon and intron numbers. The difference in exons, introns, and UTR length in the SWR1-C suggests that the subunits may have different roles to play during the growth and developmental processes of pineapple [43].

SWR1-C subunits in pineapple were further characterized based on the length of their open reading frame (ORF), protein sequence size, protein molecular weight (MW), and isoelectric point (pI) (Table 1).

### 3.2. Chromosomal Location of SWR1-C Components

**Figure 3:**
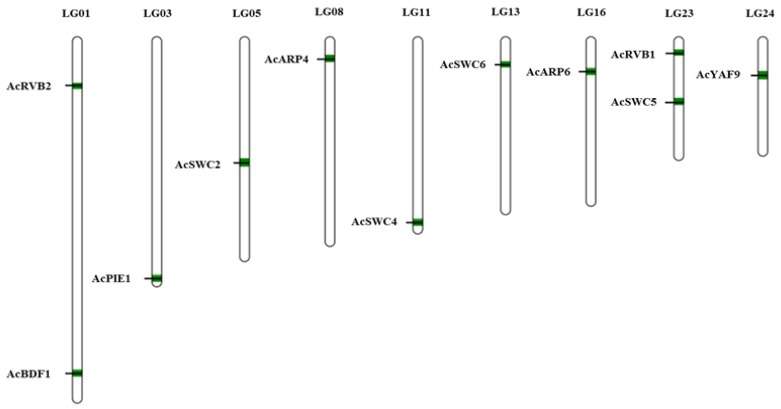
Chromosomal locations of pineapple SWR1-C subunits. The 11 SWR1 subunits of pineapple were mapped to different chromosomes using MapChart. The position of pineapple SWR1-C subunits are represented in green.

SWR1-C subunits genes were unevenly mapped on nine linkage groups (LG) out of the total 25 linkage groups present in pineapple. Based on our mapped data, we found two genes located on linkage groups 1 and 23, while linkage group 3, 5, 8, 11, 13, 16, and 24 had one gene each (Figure 3). Our data suggest that the length of the linkage group has no relation with the number of genes on it.

### 3.3. AcSWR1-C Subunits Show Differential Expression

**Figure 4:**
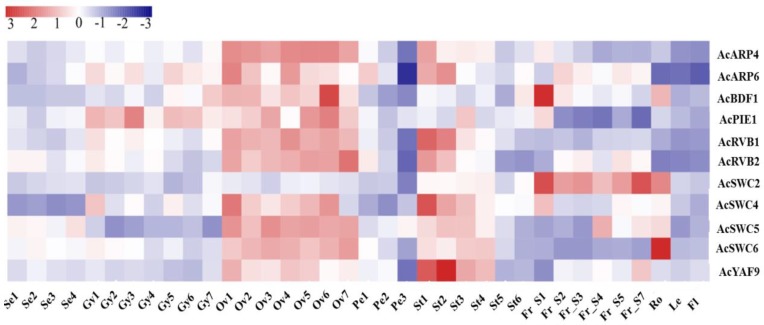
Expression profiles of pineapple SWR1-C subunits in different organs and developmental stages. Red color indicates high levels of transcript abundance, and blue color indicates low transcript abundance. The color scale is shown at top left side of the figure. Sample details are mentioned at the bottom of each lane: sepal Se1–Se4, gynoecium Gy1–Gy7, ovule Ov1–Ov7, petal Pe1–Pe3, stamen St1–St6, fruit Fr_S1–Fr_S7, root Ro, leaf Le and flower Fl. “S” is the abbreviation for “stage.” pineapple SWR1-C subunit names are shown on the right side of the figure.

Transcriptome data obtained from different developmental stages and organs was used to examine the transcriptional divergence of SWR1-C. From the RNA-Seq data and average log values of each of the pineapple SWR1-C subunits, an expression heatmap was generated (Figure 4). According to the expression data, almost all the subunits showed low expression level in the sepal except *Ac*SWC5 which was slightly more expressed. In the gynoecium, *Ac*ARP6 and *Ac*PIE1 were highly expressed, while *Ac*ARP4, *Ac*BDF1, *Ac*RVB1, *Ac*RVB2, *Ac*SWC4, and *Ac*SWC6 were less expressed. All the subunits were significantly expressed in the ovule except for *Ac*SWC2. Similarly, in the stamen, most of the subunits were highly expressed except *Ac*BDF1 and *Ac*SWC2; however, *Ac*PIE1 shows a reduced expression in stage three of stamen development. In the fruit, *Ac*ARP4, *Ac*PIE1, *Ac*RVB1, *Ac*SWC6, and *Ac*YAP9 were less expressed, while *Ac*SWC2 was expressed more compared to *Ac*ARP6, *Ac*BDF1, *Ac*RVB2, *Ac*SWC4, *Ac*SWC5, and *Ac*YAF9. This suggests that the pineapple SWR1-C subunits are differentially expressed in different organs during growth and development.

### 3.4. Expression of SWR1-C Components is Regulated Under Abiotic Stress

**Figure 5:**
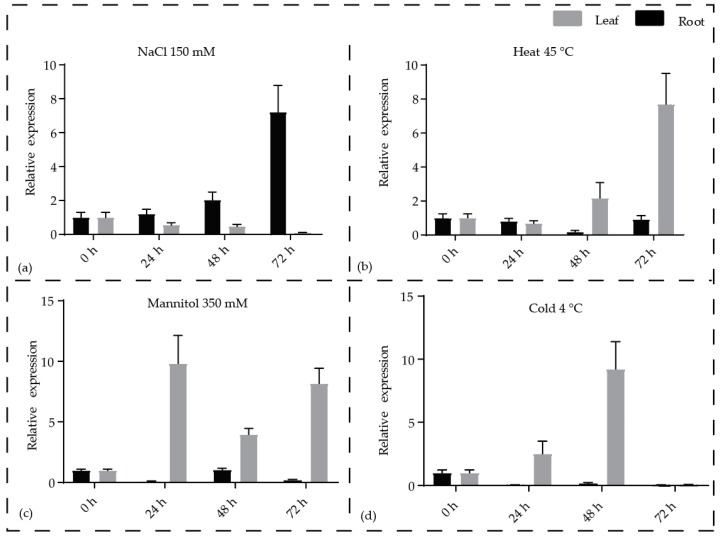
Expression profiles of the pineapple *Ac*SWC6 subunit in response to different stress treatments. qRT-PCR of *Ac*SWC6 subunit at different time point (0 h, 24 h, 48 h, and 72 h) and samples (leaf and root) after (**a**) Salt (NaCl 150 mM), (**b**) Heat (45 °C) stress (**c**) (Mannitol 350 mM), and (**d**) Cold (4 °C) stress. Data were normalized with pineapple EF1a gene and vertical bars indicate standard error (SE). All experiments were performed with three technical and three biological repeats.

The influence of abiotic stress was examined in pineapple SWR1-C on the basis of their expression during exposure to stress. All the pineapple SWR1-C subunits expression was examined by quantitative real-time (qRT) PCR under NaCl, mannitol, heat, and cold stresses, respectively. Our qRT-PCR data indicate that all the SWR1-C subunits of pineapple are regulated by the selected stress treatments. Most of the subunits showed significantly high expression to NaCl in roots, while *Ac*SWC2, *Ac*SWC5, *Ac*RVB1, and *Ac*RVB2 were significantly upregulated in both leaf and the root at different time points. All the SWR1-C subunits were significantly upregulated in the root compared to leaf under NaCl and mannitol treatments. Similarly, when subjected to heat treatment, all subunits were upregulated in leaf and the root except *Ac*SWC6, *Ac*SWC2, *Ac*SWC4, and *Ac*SWC5, which were upregulated in the leaves only. Similarly, during the cold stress, *Ac*ARP6, *Ac*BDF1, *Ac*SWC6, and *Ac*SWC2 were upregulated in the leaf, while only *Ac*ARP4 was upregulated in the root. *Ac*PIE1, *Ac*SWC4, *Ac*SWC5, and *Ac*YAF9 were less expressed in the cold treatment (Figure S1). The expression of *Ac*SWC6 was increased in the root under NaCl treatment, while it was higher in the leaf under mannitol, heat, and cold treatments when compared to the untreated plants (Figure 5a–d). Taken together, these results suggest that the SWR1-C subunits play essential functions under abiotic stress conditions.

### 3.5. AcSWC6 Gets Localized to the Nucleus

**Figure 6:**
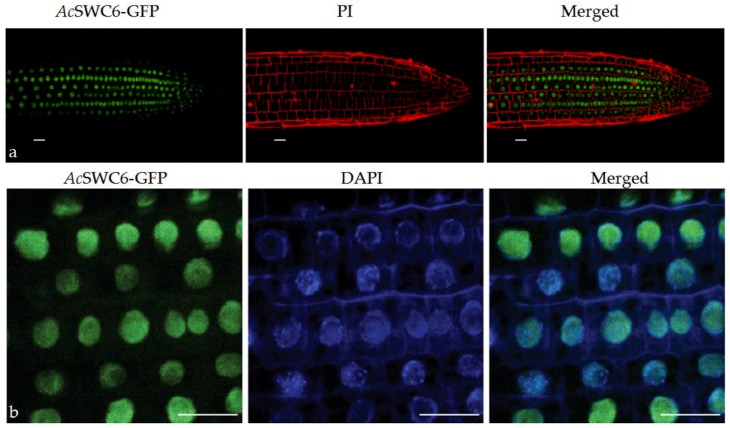
*Ac*SWC6-GFP localizes to the nucleus. (**a**) *Ac*SWC6-GFP localization in the nucleus of seven-day-old roots of transgenic *Arabidopsis* plants. GFP fluorescence is represented in green and propidium iodide (PI) fluorescence channel is represented in red (**b**) *Ac*SWC6-GFP localization in the nucleus of seven-day-old roots of transgenic *Arabidopsis* plants. GFP fluorescence is represented in green and DAPI fluorescence channel is represented in blue. Scale bars = 20 μm.

Roots of the *Ac*SWC6 transgenic plants were examined under a confocal microscope in order to understand the localization and expression of the *Ac*SWC6 protein. We found that *Ac*SWC6-GFP localizes in the nucleus of transgenic lines (Figure 6).

### 3.6. AcSWC6 Regulates the Biotic and Abiotic Stress Tolerance in Arabidopsis

**Figure 7:**
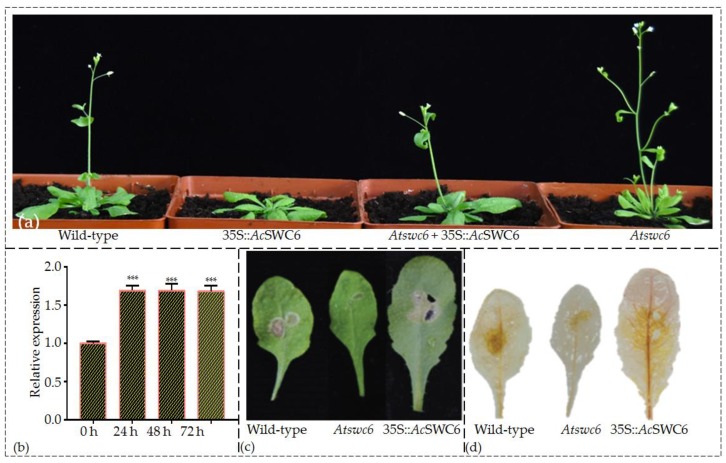
*Ac*SWC6 regulates the biotic stress tolerance in *Arabidopsis*: (**a**) Morphology of four-week-old wild-type (Col-0) and *Ac*SWC6 overexpressing plant; (**b**) Relative expression of *Ac*SWC6 in pineapple leaf at different time points after infection with fungus (*Sclerotinia sclerotiorum*); (**c**) Damage in leaves by fungus (*Sclerotinia sclerotiorum*) to mature rosette leaves of wild-type and *Ac*SWC6 overexpressing plants after 24 h infection; (**d**) Detection of H_2_O_2_ by DAB staining in leaves of wild-type (Col-0) and *Ac*SWC6 overexpressing plants after 24 h infection. The brown color reflects the reactive oxygen species (ROS) accumulation after the fungal treatment.

To examine the role of *Ac*SWC6 in immunity and pathogen resistance, we infected the leaves of the pineapple plants with the fungus *Sclerotinia sclerotiorum* and relative transcript abundance of *Ac*SWC6 was measured at different time points. We found that the fungal infection increased the *Ac*SWC6 transcript significantly (Figure 7b). This finding clearly indicates that the *Ac*SWC6 is involved in the biotic stress response in pineapple. We then checked the performance of *Ac*SWC6 overexpressing *Arabidopsis* plants compared to wild type and *Atswc6* mutant against fungal infection. Rosette leaves of the plants were incubated for 24 h to allow fungal infection. The infected leaves were then photographed (Figure 7c), followed by DAB staining (Figure 7c). The *Atswc6* mutant showed higher resistance to the fungal attack with the least damage to leaves and H_2_O_2_ activity, while the 35S::*Ac*SWC6 overexpression line was susceptible with increased damage to leaves and higher H_2_O_2_ activity compared to the mutant and the wild type (Figure 7c,d).

**Figure 8:**
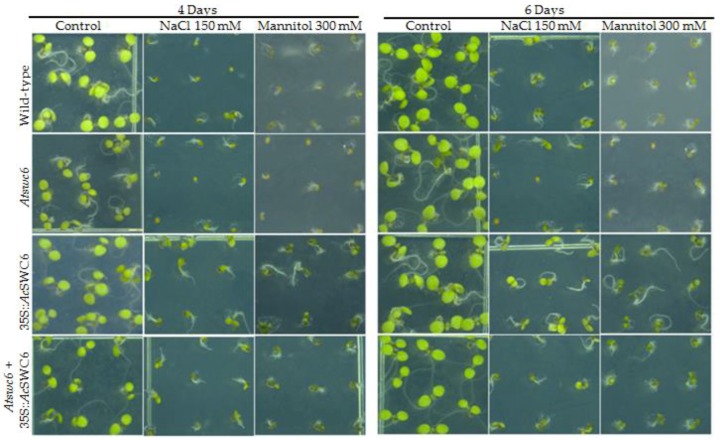
Overexpression of *Ac*SWC6 results in resistance to salinity and osmotic stress. Phenotype of 4 days and 6 days old wild type, *Atswc6*, 35S::*Ac*SWC6 and complemented line germinated under salinity (150 mM NaCl) and osmotic stress (300 mM mannitol).

Previous reports have shown that SWR1-C plays a crucial role during growth and environmental responses [6,11]. To further examine the role of *Ac*SWC6 during abiotic stress we used different *Ac*SWC6 *Arabidopsis* lines and studied their growth phenotype after 4 d and 6 d of salinity (NaCl 150 mM) and osmotic (mannitol 300 mM) stress treatment. The *Atswc6* mutant showed susceptibility to salinity and osmotic stress compared to wild-type plants. The plants ectopically expressing *Ac*SWC6 displayed better performance in terms of germination and growth phenotype during salinity and osmotic stress (Figure 8). These finding clearly indicate that the SWR1-C subunit SWC6 contributes to biotic and abiotic stress responses in plants.

### 3.7. SWC6 Function Is Conserved in Plants

**Figure 9:**
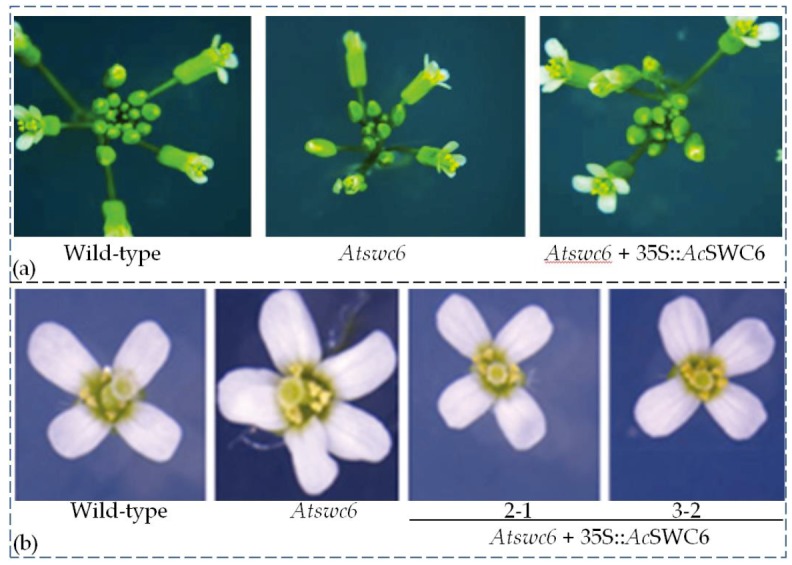
*Ac*SWC6 rescues the *Atswc6* mutant floral architecture. (**a**) Flower architecture phenotype of wild type, *Atswc6*, and *Atswc6* 35S::*Ac*SWC6 complemented lines. (**b**) Petal phenotype of wild type, *Atswc6*, and *Atswc6* 35S::*Ac*SWC6.

**Figure 10:**
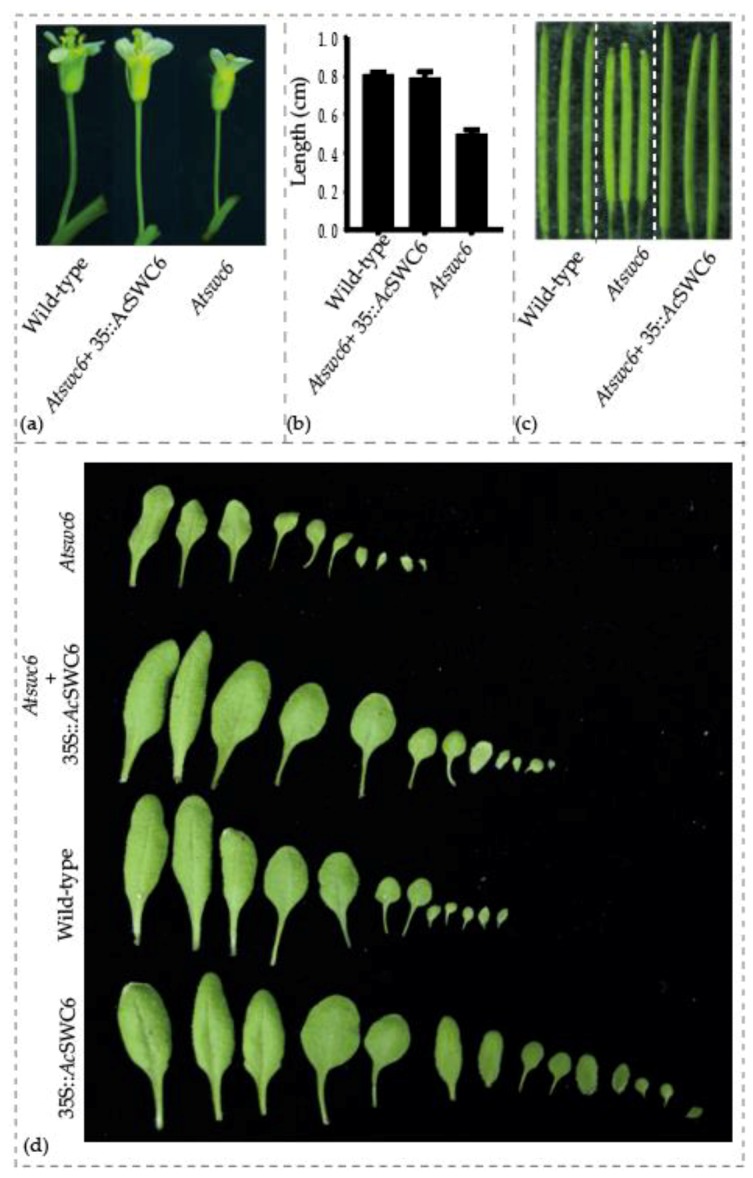
*Ac*SWC6 flower phenotype in *Arabidopsis*. (**a**) and (**b**) Pedicel length phenotype, (**c**) Silique and, (**d**) leaf phenotype of wild type, *Atswc6*, and *Atswc6* 35S::*Ac*SWC6 complemented lines.

The *Atswc6* complementation plasmid was constructed by amplifying a 5058 bp coding region of *Ac*SWC6 from pineapple genomic DNA, using primers listed in the Supplementary Table S2. It was then used to transform the *Arabidopsis swc6* mutant plants via the floral dip method. Ten different lines from the initial screening were selected for further generation advancement. Finally, plants from three independent T3 generation lines were used for phenotypic assessment. All of the transformed lines plants showed that *Ac*SWC6 can rescue the phenotype of the *Atswc6* plants (Figure 7a). There was no obvious difference in the root growth between the complemented and the wild type plants. The complemented plants exhibited a growth pattern similar to that of the wild type and the small and serrated leaves phenotype along with early flowering phenotype was rescued by *Ac*SWC6. Moreover, the small flowers and irregular floral organs phenotype, and the flower architecture phenotype were also restored (Figure 9a,b). The silique size and seed setting phenotypes were also rescued by *Ac*SWC6 (Figure 10a,c).

## 4. Discussion

Chromatin remodelers perform the task of chromatin remodeling and chromatin dynamics has significant involvement in almost every cellular activity by regulating gene expression [6,47]. Eukaryotic cells possess an evolutionary conserved ATP-dependent chromatin remodeling complex, SWR1-C, belonging to the SWI/SNF class of remodelers [48,49]. The subunits of SWR1-C have been studied in *Arabidopsis* [6,10]; however, the complex is still unexplored in pineapple. Here, we identified the subunits of SWR1-C in pineapple and studied their properties in detail. We further characterized one subunit, *Ac*SWC6, by overexpressing it and complementing the *Atswc6* mutant in *Arabidopsis*. Our results suggest the existence of SWR1-C in pineapple and a functionally conserved role of *Ac*SWC6 in plants.

In *Arabidopsis,* PIE1, ARP6, ARP4, SWC6, and SWC4 have been studied and characterized as SWR1-C subunits [6,21,50,51]. Here, we identified the homologs of the SWR1-C subunits from pineapple with high sequence similarity between that of *Arabidopsis*, rice, and yeast. The multispecies phylogenetic tree (Figure 1) revealed the conserved nature of SWR1-C between pineapple, *Arabidopsis*, and rice. The SWR1-C subunits formed the independent groups with their homologs in *Arabidopsis* and rice. Based on the phylogenetic relationship, the subunits could be further divided into three categories (Figure 2). Group I consisted of *Ac*ARP4, *Ac*ARP6, *Ac*PIE1, and *Ac*SWC5. Group II had *Ac*SWC2 and *Ac*SWC4, and Group III had *Ac*YAF9, *Ac*RVB1, *Ac*RVB2, *Ac*SWC6, and *Ac*BDF1. ACT1 was present in *Arabidopsis* and rice but not in pineapple. These findings support the existence of SWR1-C in plants. SWR1-C is expected to act as a single entity in various aspects of development and environmental cues. However, the subunits show independent function, especially in immunity and during abiotic stresses [8,10]. The possible explanation could be the presence of a high degree of exon–intron variation in the gene structure of each subunit in the complex. The final conformational structure and composition of the SWR1-C subunits affect nucleosome and H2A.Z deposition, which could influence the recruitment of transcriptional machinery [52].

We next examined the expression pattern of SWR1-C subunits in pineapple using the RNAseq data and generated a hierarchical expression map. We found that most of the SWR1-C subunits are highly expressed in the ovule and stamen except *Ac*SWC2, which shows higher expression in the fruit. This could be because SWR1-C regulates flower and gametophyte development [11]. Mutants of the core subunits of SWR1-C, PIE1, SWC6, and ARP6, exhibit defects in flowering with bushy appearance, early bolting, and defects in floral organs, indicating their involvement in floral development [19]. Among the pineapple SWR1-C subunits, *Ac*PIE1 was expressed specifically in ovule, gynoecium, and slightly in stamen stage 3, suggesting its involvement in pineapple flower development similarly indicated by *Arabidopsis pie1* mutants with severe flowering defects when compared to *swc6* and *arp6* mutants [53]. *Ac*ARP6 was expressed in the same manner as *Ac*PIE1; however, it was additionally expressed in stamen and fruit. Similarly, *Ac*SWC6 also showed additional expression in sepal, stamen, and root compared to *Ac*PIE1 (Figure 4). These results are in agreement to previous studies on the role of SWR1-C in gametophyte and flower development [5,14,22,54]. Increasing evidence suggests that SWR1-C plays a major role in plants under both optimal and stressed conditions [8,10]. We investigated the transcript level of *Ac*SWC6 under different abiotic stress conditions by qRT-PCR (Figure 5). Our data showed that the transcript level of *Ac*SWC6 changed significantly during the stress treatments. An increase in the level of the transcript of *Ac*SWC6 was noticed in the root but it decreased in the leaf during NaCl treatment (Figure 5a). However, during osmotic stress, a rise in the level of the transcript was observed in the leaf but it decreased in the root (Figure 5c). Similarly, during heat and cold stresses, an increase in the transcript level of *Ac*SWC6 was observed (Figure 5b,d). Altogether, the data suggest that *Ac*SWC6 is involved in the stress response in plants.

Plants exhibit different strategies to respond to pathogen attack and adapt or escape harsh environmental conditions due to their sessile nature [10]. SWR1-C plays a significant role in abiotic and biotic stresses by regulating gene expression during pathogen attack and environmental stress [8,10]. In *Arabidopsis*, mutation in PIE1, ARP6, and SWC6 confers resistance to pathogens [14,17]. We investigated the role of pineapple *Ac*SWC6 during biotic (fungal attack) and abiotic (salinity and osmotic) stress in *Ac*SWC6 overexpressing *Arabidopsis* plants, consistent with previous reports, we found that *Ac*SWC6 regulates the immunity of the plants during pathogen attack. The *Ac*SWC6-expressing plants resulted in pathogen susceptibility, while the *Arabidopsis swc6* mutant showed enhanced resistance to the pathogen compared to wild type and the *Ac*SWC6-expressing plant (Figure 7c,d). Additionally, during abiotic stress treatment, we found that the *Arabidopsis swc6* mutant is sensitive to salt and osmotic stress, whereas the plants ectopically expressing the pineapple SWC6 displayed increased resistance to abiotic stresses. Ectopic expression of *Ac*SWC6 may affect the deposition of H2A.Z, which in turn regulates key genes contributing to biotic and abiotic stress.

To further investigate the conserved nature of SWC6, we complemented *Arabidopsis* mutant *Atswc6* with pineapple *Ac*SWC6. The results showed that the pineapple *Ac*SWC6 could complement the *Arabidopsis Atswc6* and completely rescue the mutant phenotype. The serrated leaf, early flowering, extra petals (from 4–6), and length of the flower pedicel phenotypes were all rescued (Figure 10a–d). Flower architecture is an important determinant of reproduction in plants and it is controlled by SWR1 directly or indirectly. Pedicel elongation is controlled by the PRE1 (PACLOBUTRAZOL RESISTANCE1) bHLH transcription factor through coordination of the ER-MAPK signaling pathway and the SWR1 complex. When PRE’s expression is reduced, pedicel length is also reduced. Similarly, this phenomenon is associated with phytohormones such as brassinosteroid (BR), gibberellin (GA), and auxin. Cai et al., (2017) reported that SWR1 controls the expression of PREs and this regulates flower architecture and pedicel length [9]. Previously, Lazaro et al., (2008) reported that *At*SWC6 overexpression showed no additional phenotype [21]. However, we found that the ectopic expression of *Ac*SWC6 delayed the flowering and increased susceptibility to biotic stress (Figure 7a,c,d). This delayed flowering could result from the variation in gene structure between the SWC6 of pineapple and *Arabidopsis*. The expression of *Ac*SWC6 may cause a change in the deposition of H2A.Z, which could have resulted in the delay of flowering by regulating the expression of key genes involved in biotic and abiotic stress response.

## 5. Conclusions

In the present study, we comprehensively studied SWR1-C in pineapple and identified a total of 11 SWR1-C subunits. The expression profiles of the SWR1-C subunits in pineapple suggest that SWR1-C plays an important role in pineapple development and environmental stress response. The findings of the present study support the conserved nature and role of *Ac*SWC6 in plants during growth and development. This information could form a basis for further research to utilize SWR1-C subunits for development of next-generation crops.

## Figures and Tables

**Table 1:** Characterization of SWR1-C subunits of pineapple.

Gene	Gene ID	MW (kDa)	pI	Amino Acid	Location	ORF	Chr
***Ac*PIE1**	Aco017256	230.32	5.44	2021	16521947..16538538	6066	03
***Ac*ARP4**	Aco007686	48.75	5.09	444	1249997..1258550	1335	08
***Ac*ARP6**	Aco018334	42.98	5.97	382	2117532..2122193	1149	16
***Ac*YAF9**	Aco027612	32.38	6.72	295	2382105..2388792	888	24
***Ac*RVB1**	Aco015484	56.28	6.94	511	847770..852493	1536	23
***Ac*RVB2**	Aco012319	55.51	5.69	507	3080033..3084927	1524	01
***Ac*SWC2**	Aco016372	43.23	5.69	376	8471728..8501804	1131	05
***Ac*SWC4**	Aco005752	50.48	9.49	449	12625273..12633418	1350	11
***Ac*SWC5**	Aco007381	28.71	8.65	256	4261067..4266596	771	23
***Ac*SWC6**	Aco012501	28.51	9.03	252	1632792..1637849	759	13
***Ac*BDF1**	Aco006628	126.34	6.93	1127	23130832..23140236	3384	01

## Supplementary Materials

The following are available online at https://www.mdpi.com/2218-273X/9/8/364/s1.

## Abbreviations

SWR1	SWI2/SNF2-RELATED 1
DAB	3,3-diaminobenzidine
SWC2	SWR1 COMPLEX2
SWC3	SWR1 COMPLEX 3
SWC5	SWR1 COMPLEX 5
SWC6	SWR1 COMPLEX 6
SEF	SERRATED LEAF AND EARLY FLOWERING
SWC7	SWR1 COMPLEX 7
ARP6	ACTIN-RELATED PROTEIN 6
YAF9	YEAST ALL1 FUSED GENE FROM CHROMOSOME 9
BDF1	BROMODOMAIN-CONTAINING FACTOR 1
ACT1	ACTIN 1
ARP4	ACTIN-RELATED PROTEIN 4
SWC4	SWR1 COMPLEX 4
RVB1	RUVB LIKE PROTEIN 1
RVB2	RUVB LIKE PROTEIN 2
UTR	Untranslated region
CDS	Coding sequence
