# Supplementary material for: Identification of SWI2/SNF2-Related 1 Chromatin Remodeling Complex (SWR1-C) Subunits in Pineapple and the Role of Pineapple SWR1 COMPLEX 6 (AcSWC6) in Biotic and Abiotic Stress Response
Source: Biomolecules. 2019 Aug 13;9(8):364. doi: 10.3390/biom9080364 (PMC6723344; doi:10.3390/biom9080364)
Supplement: Supplementary file 1 [file biomolecules-09-00364-s001.pdf]

Supplementary table 1-List of Primers used for cloning and qRT-PCR

Gene Name	Gene ID	Primers
**Cloning Primers of AcSWC6**		
AcSWC6	Aco012501	FORWARD PRIMER: caccATGAATTTCGAGCATGTT
		REVERSE PRIMER: AGCAACAAACTTGAGGCA
**qPCR Primers of Pineapple SWR1c subunits**		
AcPIE1	Aco017256	Forward Primer: GCAGGCAGAAGCAGATCTTCCA
		Reverse Primer: CTGGACTGCCTTCCCTGCTAA
AcARP4	Aco007686	Forward Primer: AGGCGATGAAGTATCGGCCA
		Reverse Primer: TGATCCAACAACGGACGGAA
AcARP6	Aco018334	Forward Primer: GTTGATTCTCGCGGTGATGC
		Reverse Primer: ATCTCAGGCACAAGGAACCG
AcYAF9	Aco027612	Forward Primer: AACTGGCAGCAGCTCGTCAA
		Reverse Primer: TTGTGGCGGCCCATCAATCT
AcRVB1	Aco015484	Forward Primer: ACGCCATGAGGATCGAGGAG
		Reverse Primer: CAGCGCGGTCTTTCCTGTG
AcRVB2	Aco012319	Forward Primer: CGTCGGTCCTCACACCAAGT
		Reverse Primer: AGTGCGAGAAAGCCCTGTGT
AcSWC2	Aco016372	Forward Primer: AAGAGGAGCCAGAGGCTGTT
		Reverse Primer: TTCAGCTTCGTCATCGGGGTC
AcSWC4	Aco005752	Forward Primer: AGCACCAGGCACTCCTAAGC
		Reverse Primer: CCAGGAGCCTTGCGTTTGTG
AcSWC5	Aco007381	Forward Primer: TGCGGTTAGGGATGCCACAT
		Reverse Primer: GTCCGCGTCTACGAGCTTCT
AcSWC6	Aco012501	Forward Primer: TGCAAAGAAGGCACCCAGAA
		Reverse Primer: CGCCCTCAAATAGGTTGGGA
BDF1	Aco006628	Forward Primer: AGGCAGGTCTCCCCGGATAA
		Reverse Primer: TGTCACCCTGGTTGAGGGTC

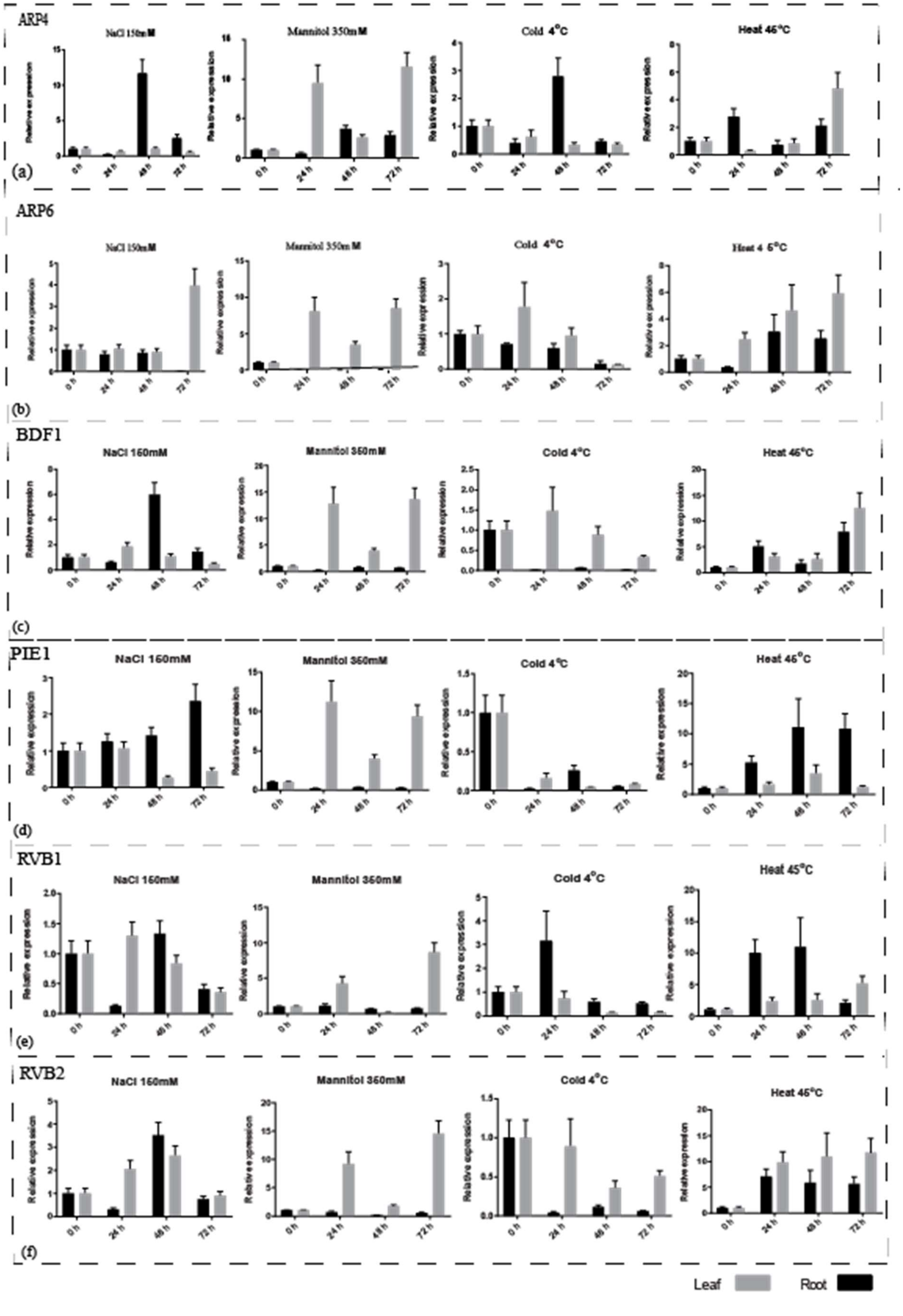

Supplementary figure 1- qRT-PCR of pineapple *Ac*ARP4, *Ac*ARP6, *Ac*BDF1, *Ac*RVB1 and *Ac*RVB2 SWR1c subunits at different time point (0h, 24h, 48h and 72h) and samples (Leaf

and root) after (a) Salt (NaCl 150 mM) (b) Cold (4°C) stress (c) osmotic (mannitol 350 mM) (d) Heat (45°C) stress

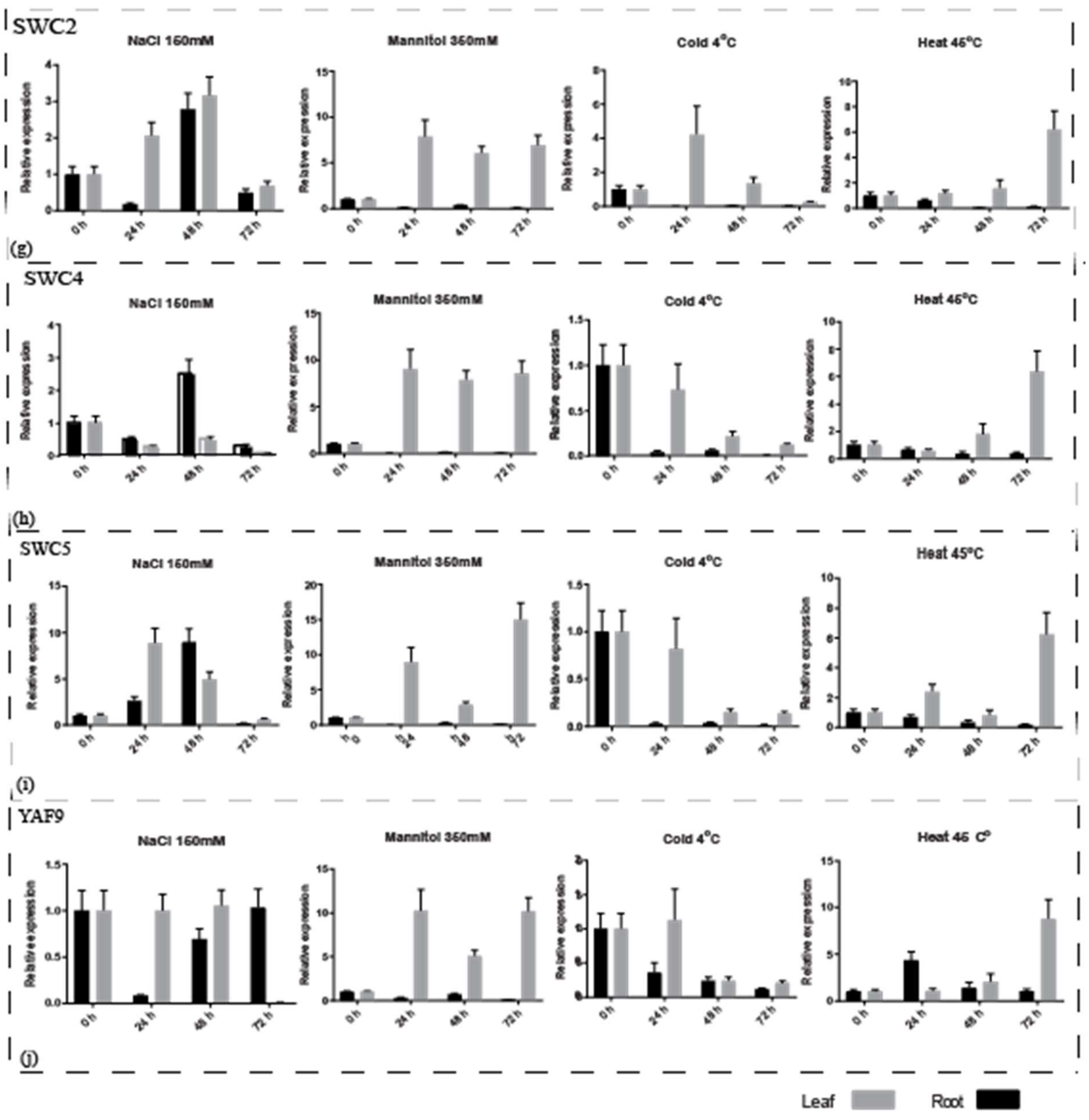

Supplementary figure 2- qRT-PCR of pineapple *Ac*SWC2, *Ac*SWC4, *Ac*SWC5, *Ac*YAF9 SWR1c subunits at different time point (0h, 24h, 48h and 72h) and samples (Leaf and root) after (a) Salt (NaCl 150 mM) (b) Cold (4°C) stress (c) osmotic (mannitol 350 mM) (d) Heat (45°C) stress.
